# Chimeric antigen receptor T-cell therapy in pediatric neurological autoimmune diseases: mechanisms, clinical applications, and future perspectives

**DOI:** 10.3389/fped.2026.1740566

**Published:** 2026-03-25

**Authors:** Weihao Ling, Chen Xu, Jihong Tang, Shifeng Lu, Chunfeng Wu

**Affiliations:** 1 Department of Neurology, Children’s Hospital of Nanjing Medical University, Nanjing, China; 2 Department of Neurology, Children’s Hospital of Soochow University, Suzhou, China; 3 Department of Hematology and Oncology, Children’s Hospital of Nanjing Medical University, Nanjing, China

**Keywords:** autoimmune diseases, CAR-T cell therapy, challenges, clinical application, pediatric neurological autoimmune diseases

## Abstract

Pediatric neurological autoimmune diseases (PNADs) are a result of immune system abnormalities that target the central and peripheral nervous systems, leading to various neurological dysfunctions in children. The limitations of current immunotherapies underscore the necessity for more efficacious treatment interventions. The objective of this review is to examine the fundamental principles, recent advancements, and clinical applications of chimeric antigen receptor (CAR) T cell therapy in the treatment of pediatric neurological autoimmune diseases (PNADs). By specifically targeting and reducing pathogenic B cells, CAR-T therapy has the potential to reset the immune system. Growing evidence from preclinical animal studies, case reports, and early clinical trials suggests that CAR-T cell therapy has therapeutic potential in managing autoimmune diseases such as myelin oligodendrocyte glycoprotein antibody-associated disease (MOGAD) and systemic lupus erythematosus (SLE). However, several challenges remain, including the high cost of treatment, safety concerns such as cytokine release syndrome and neurotoxicity, and a lack of long-term safety data in pediatric populations. Future research should prioritize optimizing the management and evaluation model for the entire process, as well as refining CAR design and development, to enhance the safe and effective clinical application of CAR T-cell therapy in patients with PNADs.

## 1 Introduction

Pediatric neurological autoimmune diseases (PNADs) refer to a group of conditions that involve abnormal/pathological immune responses that target the central nervous system (CNS), peripheral nervous system (PNS), neuromuscular junction, and muscular tissue. These disorders represent rare but increasingly recognized causes of severe neurological dysfunction in children (1–4). The potential consequences of these untreated conditions are of significant concern; approximately 60%–80% of patients may experience cognitive diminution, motor impairments, or epileptic conditions (3). The autoimmune response is characterized by the production of autoantibodies that target neuronal surface antigens; this triggers a series of physiological responses, including synaptic disruptions, breaches in the blood-brain barrier (BBB), and inflammatory responses. These mechanisms are also the ultimate cause of subsequent neurological decline and developmental challenges. Therefore, the early recognition and treatment of these conditions is vital. However, they can be difficult to diagnose because their symptoms are similar to those of other diseases. This difficulty may lead to delays in treatment.

The prevailing first- and second-line treatments, which primarily encompass immunosuppressants, achieve remission in just 40%–60% of severe pediatric cases. Moreover, refractory disease often has a poor prognosis. For example, over 40% of pediatric patients with anti-N-methyl-D-aspartate receptor (NMDA) encephalitis experience recurrence and an extended morbid trajectory of at least 2 years. Additionally, patients with neuromyelitis optica spectrum disorder (NMOSD) face irreversible disability following each episode (3, 5, 6). Chronic high-dose immunosuppression has highlighted risks, particularly among young people experiencing growth stagnation and opportunistic infections, as well as those with secondary malignancies (7, 8). Consequently, the need for effective therapies that can induce prolonged remission without inducing iatrogenic complications is evident.

The therapeutic potential of CAR-T cell therapy in modulating the immune system offers a promising approach for the management of PNADs. CAR-T cells that target B-cell markers can eliminate pathogenic B cells and plasma cells. This targets the primary problem of autoimmunity, rather than just alleviating inflammation (9, 10). The recent use of CAR-T therapy for autoimmune diseases, along with its proven success in treating oncological diseases, further supports this idea (7, 11–13). The pediatric advantages emerge in the contexts of rapid immune rebuilding capabilities, which are complemented by thymic adaptability. This suggests superior safety and efficacy. This review gives a thorough overview of the basic ideas, recent improvements, and new ways to use CAR-T cell therapy in the treatment of pediatric neurological immune diseases.

## 2 Clinical and immunological features of PNADs

Neuroimmune disorders in pediatric patients arising from autoimmune dysregulations that affect both CNS and PNS architectures encompass a myriad of conditions. Examples include autoimmune encephalitis, neuromyelitis optica spectrum disorder (NMOSD), myelin oligodendrocyte glycoprotein antibody-associated disease (MOGAD), as well as myasthenia gravis (MG) and chronic inflammatory demyelinating polyneuropathy (CIDP) (4). Studies have shown that a considerable fraction of acute syndromes characterized by encephalitis/encephalopathy or demyelination occur in younger populations. Moreover, these syndromes are associated with high rates of relapse and enduring neurological sequelae, further illustrating the propensity for necessitating prolonged immunotherapeutic intervention (1, 2).

The clinical presentation of PNADs often includes overlapping neuropsychiatric and neurological symptoms such as seizures, disruptions in cognition and behavior, motor disturbances, and altered consciousness. After the initial presentation, childhood-onset cases may exhibit these aforementioned manifestations and are often accompanied by a greater potential for relapse, with additional vulnerabilities among children raising further concerns of prolonged neurodevelopmental repercussions. These acutely insinuating symptoms may imitate infectious, metabolic, or neoplastic CNS diseases, posing a challenge to diagnostic discernment and delaying therapeutic engagement (2, 3, 14). Furthermore, the intertwining dynamics between immune maturation and neural development in pediatrics highlight the increased complexity of disease recognition and prognosis.

Within the domain of immunopathological observations, a notable proportion of these pediatric neuroimmune anomalies evidently have an established underlying basis in antibody-mediated processes that target neuronal or glial surface antigens (15). Anti-NMDAR encephalitis is a disorder that has been the subject of extensive research and is now a well-understood condition. The condition is characterized by the presence of certain antibodies that target a part of the NMDA receptor, causing it to become internalized and malfunction within the synapse (16). There are two types of disease-causing antibodies that are characteristic of demyelinating conditions such as MOGAD and NMOSD; these antibodies attack the MOG and AQP4 antigens, respectively. Additionally, the activation of complement proteins has been implicated in their pathogenesis. This activation triggers the immune system, leading to the recruitment and proliferation of immune cells. This results in further damage to glial cells such as astrocytes and oligodendrocytes (1). Younger patients may exhibit more pronounced inflammatory responses; however, they have greater capacities for tissue repair and restoration of immune homeostasis, especially with timely intervention (17, 18). These pediatric-specific mechanisms have important implications for developing advanced immunotherapeutic strategies for pediatric populations.

Standard treatment modalities for PNADs include corticosteroids, intravenous immunoglobulin (IVIG) therapy, plasma exchange, and B-cell-depleting agents, such as rituximab (4, 6). These immunomodulatory strategies have been shown to attenuate antibody-mediated inflammation to a certain extent. However, empirical studies have demonstrated that a considerable proportion of patients either fail to respond adequately or become corticosteroid-dependent, resulting in prolonged immunosuppression with increased risks of infection and impaired growth (19). These outcomes are of particular concern during critical periods of neurodevelopment (7, 8). In response to these challenges, there has been increasing focus on the advancement of innovative therapeutic interventions. These interventions have two aims: first, to improve patients’ outcomes, and second, to reduce treatment-related adverse effects. Recent studies suggest that chimeric antigen receptor (CAR)-T cell therapy has some potential as a treatment modality. This intervention has demonstrated notable efficacy in targeting and eradicating autoreactive B cells (20–23), which are responsible for the production of pathogenic antibodies. This method establishes a novel approach for immune system recalibration, thereby averting the occurrence of widespread immunosuppression.

## 3 CAR-T cell therapy: principles and development for neuroimmune targets

CAR T-cell therapy, initially developed for the treatment of hematologic malignancies, is presently being investigated for its therapeutic potential in autoimmune and neuroimmune diseases. CARs are structurally composed of an antigen-binding domain (typically an scFv), a transmembrane region, and intracellular signaling motifs that invariably include the CD3*ζ* chain and often incorporate one or more costimulatory domains such as CD28 or 4-1BB (24, 25). Following antigen recognition, T cells undergo activation, and downstream signaling cascades are subsequently triggered by the intracellular domains of their receptors. Advancements in the field of CAR design have led to the development of second- and third-generation constructs that incorporated one or more costimulatory domains. The objectives of these constructs are to enhance T-cell persistence, functional activity, and cytokine secretion. In contrast, fourth- and fifth-generation CARs incorporate safety switches or inducible cytokines to more efficiently mitigate immune responses (9, 26, 27).

Clinically, CD19-targeted CAR-T cells have demonstrated significant therapeutic efficacy in cases of treatment-refractory SLE and myasthenia gravis (MG), leading to the induction of prolonged B-cell depletion and the attainment of durable disease remission (28–31). These findings support the hypothesis that this strategy could be implemented in PNADs, where prolonged immunosuppression frequently results in significant long-term complications. A more targeted advancement of this strategy is the chimeric autoantibody receptor T cell (CAR-T), which employs autoantigen-derived extracellular domains to selectively eliminate B cells that produce pathogen-specific and disease-specific antibodies. For instance, the development of muscle-specific tyrosine kinase chimeric autoantibody receptor T (Musk-CAAR-T) cells for Musk-positive MG and desmoglein-3-CAAR-T for pemphigus vulgaris exemplifies the viability of these antigen-specific approaches in targeting autoreactive B cells (32, 33). Moreover, preliminary studies demonstrate the viability of initiating CAAR-T cells using CNS pathogens that are analogous to NMDARs. These pathogens effectively influence the development of novel treatments for autoimmune encephalitis (34).

Novel approaches in the administration methods of CAR-T therapy have also been attempted. While most applications use systemic intravenous infusion, intrathecal and intraventricular administration have been proposed to enhance CNS penetrance and reduce systemic toxicity. Preclinical models and isolated clinical cases have indicated that localized delivery may be advantageous, primarily for diseases that are confined to the CNS, such as anti-NMDAR encephalitis or MOGAD (34–36).

Nevertheless, challenges persist. CAR-T therapy has been associated with a range of risks, including cytokine release syndrome (CRS) and neurotoxicity. These potential complications could be especially problematic in children due to ongoing neurodevelopment. Moreover, long-term data in pediatric autoimmunity are limited, and challenges, such as manufacturing complexity, cost, and accessibility, hinder widespread adoption. Nonetheless, broad immunosuppression may be superseded by the ability of CAR-T and CAAR-T therapies to target autoreactive immune cells abstractly. The strategic integration of these cell-based methodologies with clinical trials holds considerable potential to yield treatments that are safer, more effective, and more durable, as our understanding of pediatric neuroimmune pathophysiology expands.

## 4 Comparison of CAR-T therapy with conventional immunosuppressive treatments

Conventional immunosuppressive therapies such as corticosteroids, IVIG, rituximab, and mycophenolate mofetil have been the cornerstone for treating PNADs (4, 37). Corticosteroids, while effective in rapidly controlling inflammation, are implicated in numerous adverse effects, including growth retardation, hypertension, and metabolic disturbances, which are particularly examined in the pediatric population (38). IVIG is generally well tolerated, but its optimal dosing and efficacy vary by patient population (1). Rituximab, targeting CD20 B cells, curtails relapse rates in disorders such as NMOSD and autoimmune encephalitis but causes prolonged B-cell depletion and impacts infection risk (39, 40). Mycophenolate mofetil inhibits lymphocyte proliferation but exhibits immunosuppression-related toxicity (4). Despite these options, many patients experience relapses or steroid dependence and merit chronic immunosuppressive therapy; however, this raises concerns about long-term neurodevelopmental and systemic adverse effects (3, 41).

CAR-T cell engineering has examined CD19, which willfully depletes autoreactive B cells responsible for pathogenic autoantibody production (42). Unlike conventional therapies, CAR-T cell therapy achieves durable remission with a limited number of infusions that resets the immune system without chronic immunosuppression (43). This antigen-specific targeting invariably supplies non-pathogenic immune cells, securing immune competence and lowering systemic toxicity (21, 44).

Nevertheless, as is shown in Table 1, CAR-T therapy has several notable limitations and risks. The high cost and complex manufacturing process of CAR-T therapy restrict widespread accessibility, especially in low-resource settings (45). Therapeutically, CAR-T therapy is associated with serious adverse events, such as CRS and immune effector cell-associated neurotoxicity syndrome (ICANS), which is associated with significant management objectives (35). CRS manifests as systemic inflammation with fever, hypotension, and organ dysfunction, often requiring immunosuppressive treatments such as tocilizumab and corticosteroids (46). Concerns about the formulation of the pediatric brain's vulnerability are supported by the finding that ICANS manifests as neurological symptoms that range from minor confusion to cerebral edema (47).

**Table 1:** Comparison table of conventional immunosuppressive treatments and CAR-T therapy for PNADs.

Comparison dimension	Conventional immunosuppressive treatments	CAR-T therapy
Feasibility	Widely accessible; easy to administer via oral or intravenous routes; established processes.	Limited accessibility; complex manufacturing; requires specialized facilities and teams.
Efficacy	Corticosteroids offer rapid inflammation control but with variable efficacy overall; high relapse and steroid-dependence rates; chronic use required.	Antigen—specific autoreactive B-cell depletion targeting CD19; durable remission; limited number of treatments; preserves immune competence.
Risks	Corticosteroids cause growth retardation, hypertension, and metabolic disturbances; rituximab increases infection risk; long—term neurodevelopmental and systemic toxicity. IVIG can cause hypotension, allergic reaction, hives, chest pain, thrombosis, and aseptic meningitis, with the risk increased in IgA-deficient patients. Plasma exchange often must be performed in the intensive care unit, with the risk of hypotension, coagulopathy, line-related infection or thrombosis and electrolyte disturbances.	Severe acute events including cytokine release syndrome with fever and hypotension, and immune effector cell—associated neurotoxicity syndrome with symptoms from confusion to cerebral edema; higher risk for pediatric brain; intensive adverse event management needed.
Pharmacoeconomics	Low per-treatment cost for corticosteroids; high long-term costs due to chronic use and adverse event management; high per-infusion cost for IVIG.	Extremely high upfront cost due to complex manufacturing; potential long-term savings if durable remission is achieved.
Long-term Prognosis	Long-term outcomes vary due to relapses, steroid dependence, and long-term damage; impaired immune competence with chronic use.	Limited pediatric data; ongoing trials on remission durability and late toxicities; theoretical reduction in long-term damage.

Furthermore, data on CAR-T therapy's long-term efficacy and safety in pediatric autoimmune disorders are also limited, as most available evidence are from oncology and adult autoimmune cohorts (19, 28, 36). Optimal dosing regimens, patient selection, the viability of remission, immune reconstitution, and late toxicities are being studied in ongoing clinical trials in pediatric populations (48). Ethical issues regarding informed consent and potential long-term risks must also be addressed in this vulnerable group (24).

## 5 Current evidence of CAR-T application in pediatric neuroimmune diseases

CAR-T cell therapy for autoimmune diseases, such as SLE and autoimmune cholangitis, was initially validated in murine models. Murine anti-CD19 CAR-T cells with CD28 or 4-1BB costimulatory domains prevent disease onset and progression in MRL-lpr mice (49). PD-1-targeted CAR-T therapy reduces CD8 + tissue resident memory cells in the livers of mice with autoimmune cholangitis, thereby lowering bile duct inflammation (50). In a previous study, NMDAR and MuSK-CAAR T cells were administered in mouse models of NMDAR-associated autoimmune encephalitis and myasthenia gravis, respectively, resulting in reduced antibody levels and facilitating phase 1/2 clinical trials (33, 34). Recently, preliminary evidence derived from case reports and pilot trials underscores the promising therapeutic potential of chimeric antigen receptor T-cell (CAR-T) therapy in managing autoimmune diseases across both adult and pediatric populations. Key studies are further summarized in Table 2.

**Table 2:** Outcomes of select early clinical trials and compassionate use of CAR-T therapy in adults and children with autoimmune diseases.

Disease	Patients, gender/age	CAR target(s)	Study type	Summary of outcomes	Reference
SLE	F/20, M/23, F/22, F/24, F/18, F/38, F/33, F/35	CD19	Clinical trial	All participants achieved drug-free remission with autoantibody clearance and sustained remission over 29 months.	(41)
IIM	M/41, F/43, M/42				
SSc	M/60, M/36, F/37, M/47				
SLE with lupus nephritis	F/15	CD19	Case report	The patient discontinued dialysis with the SLEDAI-2 K score dropping from 23 to 0, and remained off immunosuppressants for over 12 months	(85)
SLE	F/12, F/12	CD19	Case report	Both 2 patients stopped the use of all immunosuppressants with their SLEDAI-2 K score decreased significantly respectively from 12 to 0 and 12 to 4	(31)
MOGAD	M/18	CD19	Case report	The patient remained free of relapses without immunotherapy with MOG-IgG titers undetectable since CAR-T cell infusion for 1 year	(51)
MG	F(n) = 10, M(n) = 4, mean age=52	BCMA	phase 1b/2a	Totally 14 participants’ MG-ADL, QMG, MGC, and MG-QoL-15r showed improvement, with no dose-limiting toxicity, CRS or neurotoxicity.	(28)
AQP4-IgG seropositive NMOSD	F(n) = 10, M(n) = 2, mean age=48	BCMA	phase 1	Totally 11 participants remained relapse-free, with serum AQP-4 antibodies showing a downward trend and reported improvements in disability and quality-of-life outcomes during a median follow-up of 5.5 months.	(19)

Studies in this table were identified through a targeted literature search of PubMed (2021–2025) and ClinicalTrials.gov (accessed on October 1, 2025). We included early-phase clinical trials, case series, and compassionate-use reports that reported clinical outcomes of CAR-T therapy in autoimmune diseases. PubMed was searched with the strategy consisted of title/abstract key words and subject headings to describe the key concepts of “chimeric antigen receptor T-cell therapy”, “CD19-Directed CAR T-Cell”, “Anti-BCMA”, “autoimmune disease” and “immunologic”. ClinicalTrials.gov was searched using Condition/disease = “Autoimmune Diseases of the Nervous System”; Intervention/treatment = “CAR-T Cell Therapy”; Study status = “All studies.” CAR- T; chimeric antigen receptor T cell, CAR; chimeric antigen receptor, SLE; systemic lupus erythematosus, IIM; idiopathic inflammatory myositis, SSc; systemic sclerosis, MOGAD; myelin oligodendrocyte glycoprotein antibody-associated disease, MG; myasthenia gravis; SLEDAI-2 K; Systemic lupus erythematosus disease activity index 2000, BCMA; B-cell maturation antigen, MG-ADL; MG Activities of Daily Living, QMG; Quantitative MG, MGC; MG Composite, MG-QoL-15r; MG Quality of Life 15-revised.

Clinical trials of autologous CAR-T therapy in pediatric patients for rheumatic diseases such as systemic lupus erythematosus (SLE), Sjögren's syndrome (SS), dermatomyositis, Still's disease, rheumatoid arthritis, and vasculitis are currently ongoing with early promising results. A search of ClinicalTrials.gov (accessed on October 1, 2025) identified 24 clinical trials investigating CAR-T cell therapy as a primary intervention for autoimmune diseases in pediatric populations, five of which specifically targeted PNADs (Table 3). Although CAR-T for CNS autoimmune diseases in children is still in early stages and faces challenges, including the discontinuation of a phase 1 trial for AQP4-IgG-positive NMOSD due to recruitment issues, the number of studies in this area is growing steadily.

**Table 3:** A summary of clinical trials of CAR-T cell therapy conducted in pediatric patients with autoimmune diseases.

Therapeutic indications	CAR target(s)	NCT number	Phase	Ages eligible	Status	Number	Locations
Refractory autoimmune diseases	CD19	NCT06688799	Phase 1/2	Child, adult, older adult	Recruiting	18	Beijing GoBroad Hospital
SLE	CD19/BCMA	NCT06222853	Early Phase 1	Child, adult, older adult	Recruiting	9	Zhejiang University
LN	NA	NCT06904729	Phase 3	Child, adult	Recruiting	50	Guangzhou Women and Children's Medical Center
SLE/LN	CD19	NCT06585514	Phase 1/2	Child, adult, older adult	Recruiting	18	Beijing GoBroad Hospital
SLE	BCMA/CD70	NCT06934447	Phase 1	Child, adult, older adult	Recruiting	18	The Children's Hospital of Zhejiang University School of Medicine
SLE	CD19	NCT06465147	Phase 1	Child, adult	Recruiting	12	Seattle Children's Hospital
SLE/LN	CD19-Targeted NEX-T	NCT07015983	Phase 2	Child, adult, older adult	Not yet recruiting	89	Juno Therapeutics, Inc., a Bristol-Myers Squibb Company
SS	CD19/BCMA	NCT05085431	Early Phase 1	Child, adult, older adult	Recruiting	9	Zhejiang University
Scleroderma	CD19/BCMA	NCT05085444	Early Phase 1	Child, adult, older adult	Recruiting	9	Zhejiang University
Refractory ANCA-AAV	CD19	NCT06508346	NA	Child, adult	Recruiting	12	The Children's Hospital of Zhejiang University School of Medicine
ANCA-AAV/IIM/SSc/SLE	NA	NCT06308978	Phase 1	Child, adult, older adult	Recruiting	224	Fate Therapeutics
Refractory SLE-LN/SSc/pSS-PAH	CD19/BCMA	NCT06947473	Phase 1/2	Child, adult, older adult	Not yet recruiting	45	Beijing GoBroad Hospital
DM/Still's disease/CD/UC	CD7	NCT05239702	Early Phase 1	Child, adult, older adult	Recruiting	75	Zhejiang University
ITP	NA	NCT06352281	Phase 1/2	Child, adult, older adult	Recruiting	10	920th Hospital of Joint Logistics Support Force of the People's Liberation Army of China
AHA	CNCT19	NCT06231368	Phase 1	Child, adult, older adult	Active, not recruiting	6	Institute of Hematology & Blood Diseases Hospital, China
IIM/DM/ ASyS/ IMNM/JIIM	CD19	NCT06154252	Phase 1/2	Child, adult, older adult	Recruiting	24	Cabaletta Bio
POEMS/Amyloidosis/AHA/Vasculitis	CD19/BCMA	NCT05263817	Early Phase 1	Child, adult, older adult	Recruiting	75	Zhejiang University
Relapsed and/or refractory AQP4-IgG Seropositive NMOSD	CD19/20	NCT03605238	Phase 1	Child, adult, older adult	Withdrawn	0	Chinese PLA General Hospital

SLE; systemic lupus erythematosus, BMCA; B-cell maturation antigen, LN; lupus nephritis, SS; Sjogren's syndrome, ANCA-AAV; anti-neutrophil cytoplasmic antibody associated vasculitis, IIM; idiopathic inflammatory myositis, SSc; systemic sclerosis, pSS-PAH; primary Sjogren's syndrome with pulmonary hypertension, DM; dermatomyositis, CD; Crohn disease, UC; ulcerative colitis, ITP; idiopathic thrombocytopenic purpura, AHA; autoimmune hemolytic anemia, ASyS; anti-synthetase syndrome, CNCT-19; chimeric neoantigen cell therapy-19, IMNM; immune-mediated necrotizing myopathy, JIIM; juvenile idiopathic inflammatory myopathy, NA; not available, NMOSD; neuromyelitis optica spectrum disorders.

Emerging real-world clinical evidence from the novel compassionate use of CAR-T therapy in pediatric and young adult patients supports its efficacy and safety. A previous study reported the case of an 18-year-old patient with MOGAD who remained MOG IgG positive with two episodes of myelitis and six attacks of optic neuritis within a 6-year period. Due to the aggressive progression of the disease and the presence of severe visual impairment despite multiple immunotherapies, CAR T-cell therapy was approved for compassionate use. Since receiving CD19-directed CAR T-cell infusion, the patient had not experienced CRS and ICANS. He eventually achieved drug-free remission and was MOG IgG negative after 1 year of follow-up (51). In another study, a 12-year-old pediatric patient with severe, refractory juvenile dermatomyositis, who was unresponsive to multiple immunosuppressants including rituximab, received autologous second-generation anti-CD19 CAR T cells. CAR T cells expanded and achieved complete B cell depletion. The patient experienced mild CRS, transient anemia, and neutropenia but did not have infections or neurotoxicity. Notably, skin and muscular disease activity showed remarkable, sustained improvement even after B cell recovery (52). Xue He et al. also reported two pediatric cases of refractory SLE with disease recurrence within 3 years despite multiple immunosuppressive agents, who were treated by CAR-T therapy. B cells were fully depleted and reconstituted by day 60. Grade 1 CRS and ICANS resolved with low-dose glucocorticoids. Complement C3 levels normalized by day 28, while immunoglobulin G levels normalized by day 60. SLEDAI-2K scores dropped significantly during follow-up (Table 4) (31).

**Table 4:** Efficacy and safety of CAR-T cell therapy in two pediatric patients with refractory systemic lupus erythematosus.

Characteristic	Patient 1	Patient 2
Sex	Female	Female
Age	12	12
Disease duration	>3 years	>4 years
Clinical manifestations	Recurrent skin rashes, ulcers, arthritis, and macrophage activation syndrome	Hypertension, pleurisy, unmanageable hematuria, and proteinuria
Previous medication history	Glucocorticoids, ciclosporin, mycophenolate mofetil, hydroxychloroquine, belimumab	Glucocorticoids, hydroxychloroquine, cyclophosphamide, mycophenolate mofetil, enalapril, rituximab, belimumab, tacrolimus
Renal biopsy	NA	Class IV lupus nephritis with an activity index of 11/24 and a chronicity index of 1/12
SLEDAI-2K score before Treatment	12	12
CAR-T cell count administered intravenously	1 × 10^5^/kg	
Peak time of CAR-T cell after administration	Days 7–10	
B cell depletion	Day 7	
B cell reconstitution	Day 60	
CRS	Grade 1	
ICANS	Grade 1	NA
SLEDAI-2K score at follow-up	0	4
Improved renal biopsy findings	NA	Class IV lupus nephritis with an activity index of 2/24 and a chronicity index of 5/12
Anti- dsDNA Ab level at follow-up	Negative	Returned to normal

CAR-T; chimeric antigen receptor T cell, SLEDAI-2 K; systemic lupus erythematosus disease activity index 2000, CRS; cytokine release syndrome, ICANS; immune effector cell-associated neurotoxicity syndrome, Ab; antibody.

Children show faster immune reconstitution due to higher thymic activity and immune plasticity. In trials for pediatric CNS disorders, such as diffuse intrinsic pontine glioma (DIPG) treated with B7-H3 CAR-T, children achieved rapid B-cell depletion and long-term remission, with a median survival of approximately 19.8 months compared to ≤11 months in untreated adults (36). Although CRS in pediatric patients is typically mild (Grade 1–2) and manageable with tocilizumab, the risk of neurotoxicity varies. Pediatric patients exhibit increased vulnerability to ICANS and necessitate close surveillance (46, 53, 54). Notably, CAR-T may worsen long-term cognitive impairment in children by affecting microglial activity and hippocampal neurogenesis; hence, close monitoring is required (55). Among the reported cases, B-cell reconstitution occurs more rapidly in children, typically within 3 months, compared to over 100 days or more in adults (31, 56); this rapid recovery may reduce the infection risk but increase the risk of autoimmune relapse. This also emphasizes the need for age-specific infection prevention and vaccination strategies. After CAR-T, immune markers must be closely tracked due to children's immature immunity. Their developing brains also require extended cognitive assessments beyond standard oncology tools (57). A clinical study is currently ongoing to evaluate potential cognitive effects, such as its effects on thinking, processing, and memory, of CAR T-cell therapy-induced neurotoxicity in children and young adults (NCT05237986).

## 6 Treatment workflow, adverse effects, and safety management

**Figure 1:**
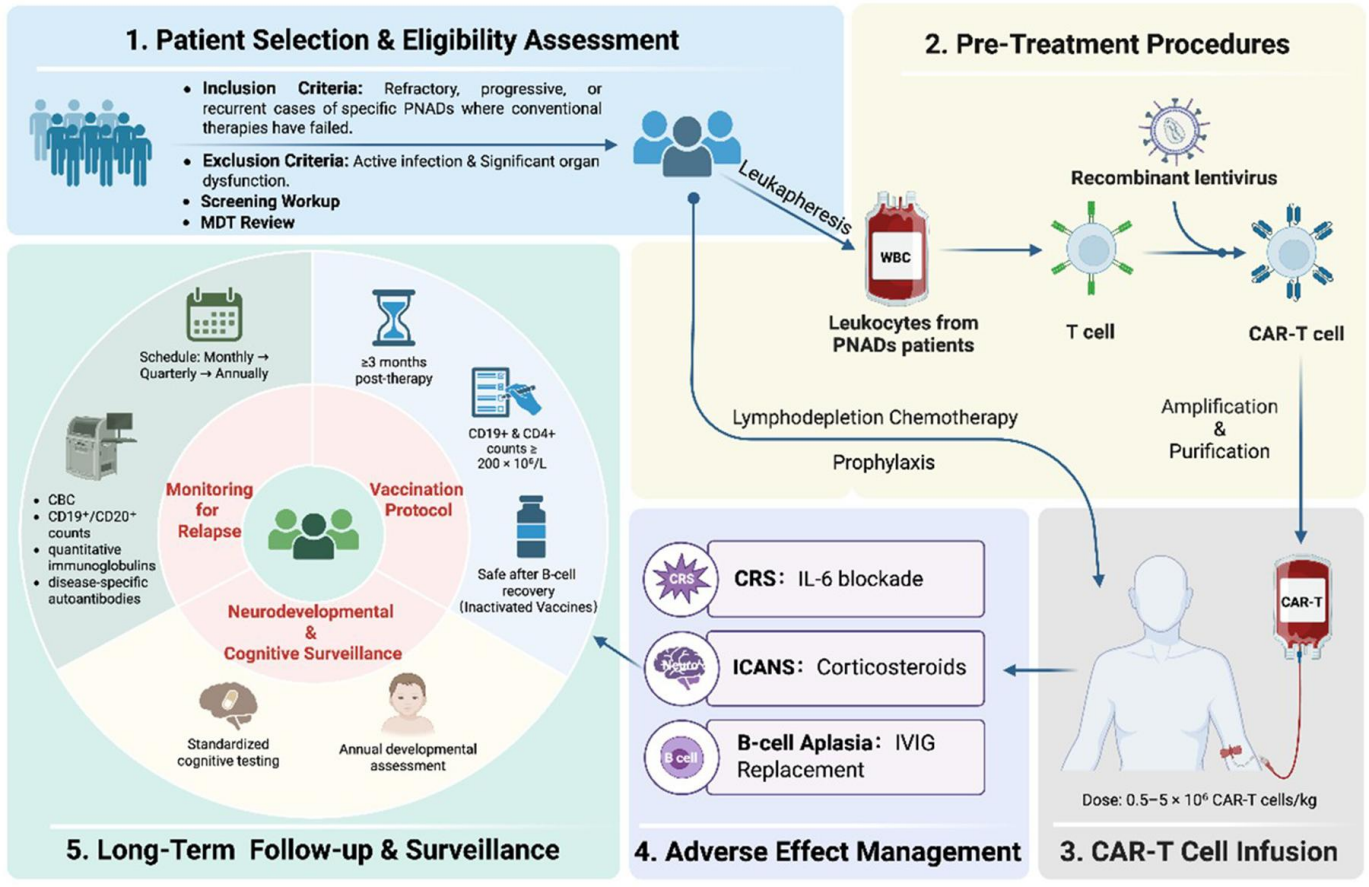
A proposed comprehensive management framework for CAR-T cell therapy in patients with pediatric neurological autoimmune diseases (PNADs). A comprehensive management framework for CAR-T therapy in PNADs is outlined, covering patient selection for refractory cases, pre-treatment procedures (leukapheresis, lymphodepletion, CAR-T production), administration of low-toxicity dosing, management of adverse effects (CRS, ICANS, B-cell aplasia), and implementation of long-term follow-up for relapse, vaccination, and neurodevelopmental monitoring.

A standardized workflow for CAR-T therapy in PNADs has yet to be established. Nonetheless, there are already established clinical guidelines for the utilization of this treatment in children with refractory CD19 + acute lymphoblastic leukemia (24). Ongoing research continues to explore the applications of CAR-T in autoimmune diseases of the central nervous system across both adult and pediatric populations (58). Therefore, a comprehensive management framework for pediatric CAR-T therapy is required, with particular emphasis on treatment workflow, adverse effects, and safety management (Figure 1).

Pediatric patients with refractory, progressive, or recurrent neuroimmune disorders, including MS, NMOSD, MOGAD, and some subentities of autoimmune encephalitis (AE), such as NMDA receptor encephalitis, who meet the diagnostic criteria for the aforementioned syndromes and have no evidence of active infection or organ dysfunction, may be considered for preconditioning (24, 37). Screening tests, including hematology tests, bilirubin levels, electrolyte levels, creatinine clearance, infectious disease testing, cardiac function, and CNS imaging, should be conducted on patients before leukapheresis. Decisions on personalized bridging treatment should be made by a multidisciplinary team following review of response to prior therapy, overall tumor burden, and disease location, as tumor presence may drive paraneoplastic autoimmunity, influence treatment choices and prognosis (3, 58). Recent studies show that CAR-T leukapheresis can be effective in pediatric patients with low absolute lymphocyte count (ALC), despite the recommended threshold of 0.2 × 10⁹/L (54).. CAR-T lymphodepletion (fludarabine 30 mg/m^2^/day + cyclophosphamide 300 mg/m^2^/day ×3 days) achieves lymphocyte nadir 48–72 h pre-infusion (31). Additionally, oral administration of sulfamethoxazole and trimethoprim has been recommended as a prophylactic measure against *Pneumocystis jirovecii* infection. CAR-T dosing (0.5–5 × 10⁶ CAR T cells/kg; lower than oncology protocols) uses a conservative fractional initial infusion to mitigate toxicity (7, 21). Acute monitoring necessitates ≥14 days of hospitalization for CRS/ICANS surveillance (54).

CRS occurs in >75% of pediatric CAR-T autoimmune recipients, distinctively expanding within 14 days post-infusion (54, 59). Over 80% of cases exhibited mild to moderate severity (Grade 1–2: experiencing symptoms such as fever and fatigue), while less than 5% were classified as severe (Grade ≥3: characterized by hypotension and/or organ dysfunction) (8, 59, 60). Notably, severity is epitomized in autoimmune cases vs. oncology cohorts. First-line management employs IL-6 blockade (tocilizumab 8 mg/kg) (8, 24, 25). ICANS, rated in severity using American Society for Transplantation and Cellular (ASTCT), presents with encephalopathy, aphasia, seizures, and rare cerebral edema, occurs in <15% pediatric patients, and typically surges on days 3–5, often requiring more aggressive neuroprotection compared to that for CRS management. Children exhibit heightened neurodevelopmental vulnerability due to incomplete myelination of white matter tracts, increasing susceptibility to ICANS-related neural injury. This structural immaturity predisposes them to atypical neurotoxicity manifestations such as paraparesis, distinct from cytokine-driven adult ICANS. Therefore, baseline observations and neurological assessments, including the Cornell Assessment of Pediatric Delirium (CAPD), are essential and are typically conducted twice daily (54, 56, 57, 59). Moreover, neuroimaging may reveal abnormalities in Grade 3–4 with focal/diffuse cerebral edema. Abnormal and prominent signals in the dorsal columns of the spinal cord have been observed in children and adolescents with quadriplegia and paraplegia following CAR-T therapy, as shown by neuroimaging (61). Key interventions for ICANS include a short course of corticosteroids (dexamethasone), ICU admission to ensure airway safety, neurology involvement for seizures (consisting of levetiracetam as a preventive measure), and an electroencephalography (EEG) has marked non-convulsive status epilepticus (54, 59, 62). Delayed neurotoxicity is rare; however, it remains a critical concern that must be promptly reported and carefully managed. To address these issues, pediatric neuropsychological monitoring must integrate standardized cognitive testing such as Wechsler or Behavior Rating Inventory of Executive Function (BRIEF) at 0, 6, 12, and 24 months, electrophysiological surveillance such as EEG during the critical risk phase of ICANS, and annual developmental assessments (63, 64).

B-cell aplasia typically persists for several months in children, with 25% of cases lasting for up to 12 months, which correlates with heightened infection risk during the first year (60, 65). IVIG replacement (0.4 g/kg monthly) is initiated for hypogammaglobulinemia (<4 g/L) with serious or recurrent/chronic infections in children until B-cell reconstitution and immunoglobulin levels reach age-adapted normal ranges (54). The commencement of vaccination adheres to strict protocols. It is recommended that patients receiving CAR T-cell therapy wait for a minimum of 3 months before receiving routine vaccines. Additionally, it is essential to ensure that both CD19 + and CD4 + cell counts are ≥200 × 10⁶. Inactivated vaccines are regarded as safe and can be resumed post-B-cell recovery, while live attenuated vaccines are deferred for at least 6–12 months due to theoretical viral reactivation risks. The resumption of immunization is contingent upon the fulfilment of rigorous criteria, including documented evidence of immune recovery, a prior response to inactivated vaccines, and the cessation of immunoglobulin therapy (54, 66–69). Late-effect surveillance include monitoring for autoimmunity reactivation. The follow-up schedule progresses from monthly to quarterly, then annually, with lab monitoring such as Complete Blood Count (CBC), CD19^+^/CD20^+^ counts, quantitative immunoglobulins, and disease-specific autoantibodies to detect subclinical relapse (54, 67).

## 7 Challenges, ethical considerations, and future perspectives

Nonetheless, CAR-T therapy is demanding for pediatric patients due to multiple factors. Economic and resource constraints have considerably limited access to pediatric CAR-T therapy, as the total treatment costs is approximately $500,000 per patient. The 2–4-week autologous manufacturing timeline bears the risk of clinical deterioration in acute neuroimmune conditions, such as anti-NMDAR encephalitis, where 40% of pediatric patients consolidate irreversible neurological disability during production delays (3, 45, 70). Successful implementation furthermore requires specialized infrastructure, including apheresis units, GMP-compliant laboratories, pediatric neuro-intensive care units, and multidisciplinary clinical teams, available at only a few medical centers.

Pediatric CAR-T therapy also involves major ethical challenges. Given that CAR-T therapy is currently primarily in the clinical trial stage for children with neuroimmune diseases, ethical considerations mainly include informed consent, along with risks/benefits, which vary considerably between patients. Since children cannot independently make medical decisions, surrogate consent must be paired with age-appropriate assent methods that include full disclosure of long-term risks, many of which may be difficult for guardians to fully understand (71). This highlights the need for mandatory ethics committee review beyond standard oncology procedures. Long-term monitoring also creates ethical obligations, as institutions must follow up patients for at least 15 years to evaluate delayed neurocognitive effects, immune issues, fertility risks, and potential cancer risks, which is considerably longer than that of most clinical trials (48). Most importantly, most children in low- and middle-income countries (LMICs) cannot access CAR-T due to high costs and lack of infrastructure, further limiting its availability to high-income regions (70). Addressing this disparity may require the intervention of international organizations, as exemplified by the collaboration between the World Health Organization (WHO) and the European Society for Medical Oncology (ESMO) in the field of cancer control (72).

The manufacturing process of CAR-T cells is continually being refined through innovation, thereby expanding their functional capabilities in next-generation CAR designs to meet diverse clinical challenges. The second-generation CAR structure is achieved by adding a costimulatory signal domain of CD28 or 4-1BB, while the third-generation CAR structure combines CD28 and 4-1BB or CD28 and OX-40 to exhibit different characteristics. These have contributed to the notable efficacy of CAR-T cells in treating oncological diseases and certain autoimmune disorders (25, 27, 73, 74). The fourth- and fifth-generation CAR T-cells are both designed based on predecessors, featuring a diversified group of CAR T constructs and a novel co-stimulatory domain, which can enable more precise targeted therapy and support their growth and persistence. These two therapeutic approaches are yet to be used in preclinical *in vivo* studies (75). In particular, RNA-based CARs utilize transient mRNA expression to address the unregulated CAR T-cell proliferation, offering enhanced safety for exploratory applications in neuroimmune diseases by limiting off-target toxicity (28), which is particularly valuable for pediatric first-in-human trials. Off-the-shelf allogeneic CAR T-cell therapies can reduce overall costs by removing patient-specific apheresis, reducing logistics expenses, and increasing production scalability to address critical barriers in acute pediatric settings (76). Dual-targeting CARs such as CD19/BCMA mitigate antigen escape risks. Numerous phase I clinical trials employing this approach for the treatment of autoimmune diseases in both adult and pediatric populations are currently in progress (7). Armored CARs that secrete IL-7 and IL-15 or other factors, as well as switchable CARs whose activity is regulated by small molecules, have been shown to have enhanced tumor microenvironment penetration and facilitate real-time toxicity management. These constructs are currently undergoing Phase I/II clinical evaluation in children with different types of cancers (77, 78). Collectively, these innovations are designed to enhance safety and improve treatment accessibility for pediatric patients with neuroimmune diseases.

Moreover, antigen-specific precision engineering now enables the development of patient-tailored CAAR-T cell therapies targeting pathogenic autoantibodies. GluN1-directed CAAR-T effectively eliminates autoantibody-producing B cells in anti-NMDAR encephalitis, AQP4-CAAR-T achieves antigen-specific B-cell depletion in NMOSD, and MUSK-CAAR-T is undergoing preclinical validation for MG (34, 42). Advancing the treatment timeline of CAR-T intervention is increasingly considered for children with refractory autoimmune diseases such as refractory SLE. Administering CAR-T during clinical remission helps to re-establish immune tolerance, prevent neurodegenerative damage, and reduce relapse rates (74). Biomarker-integrated protocols utilize serum and CSF antibody titers such as CSF anti-NMDAR antibody in anti-NMDAR encephalitis and serum MOG-IgG in MOGAD, serum neurofilament light chain levels in MS, and cytokine profiles such as increased CXCL13, indicating B-cell activity to inform treatment timing, dosing, and early intervention strategies (79–82). Future therapeutic frameworks might aim to integrate these biomarkers with *in vivo* CAR-T cell tracking via FDG-PET/MRI for real-time precision monitoring (83, 84).

## 8 Conclusion

CAR T cells are genetically engineered T cells that express chimeric antigen receptors composed of an scFv antigen-binding fragment, a hinge, a transmembrane domain, and one or more intracellular signaling or co-stimulatory domains to recognize cell-surface antigens. Upon antigen binding, they proliferate and release cytotoxic molecules to kill multiple target cells. Next-generation CARs include co-stimulatory domains to enhance activation, expansion, and persistence. This provides a clear mechanistic foundation and a feasible targeted cellular therapy approach for the treatment of PNADs.

CAR-T cell therapy signifies a significant advancement in the treatment of refractory PNADs, potentially providing long-term, drug-free remission through the targeted elimination of pathogenic autoreactive B cells that drive specific antibody production. This mechanism supports the concept of an “immune reset” in some contexts, which contrasts with conventional broad-spectrum immunosuppressive therapies that lack specificity and are associated with long-term toxicity risks, particularly in children undergoing critical developmental phases. Preclinical animal studies initially confirmed the effectiveness of this treatment approach. Early clinical trials of adult neurological autoimmune diseases such as MG and AQP4-IgG-positive NMOSD have also yielded promising results. Early trials and compassionate use programs in adolescents and children have shown encouraging safety and efficacy profiles, particularly in cases of MOGAD and refractory SLE. These findings highlight the potential of CAR-T therapy to fundamentally improve treatment outcomes in pediatric severe autoimmune diseases.

Nevertheless, the integration of CAR-T therapy into the management of PNADs poses considerable challenges. It is imperative to acknowledge paramount safety concerns, including but not limited to CRS and ICANS. These concerns entail meticulous observation and formulation of targeted neuroprotective interventions. Prolonged B-cell depletion requires comprehensive infection prevention strategies, immunoglobulin replacement therapy during immune reconstitution, and careful structured vaccination schedules. Moreover, access to CAR-T therapy is restricted by the high cost, and the intricacy of the manufacturing and delivery processes, alongside stringent infrastructure requirements.

Overall, rigorous pediatric clinical trials and the implementation of multidisciplinary care models have been identified as imperative to harnessing the transformative potential of CAR-T therapy in PNADs. These initiatives are of pivotal importance in securing access to durable, efficacious therapies for children with severe neuroimmune disorders. The overarching objective of these initiatives is to improve long-term health and neurodevelopmental outcomes. Future advancements in this field are anticipated to prioritize enhancing the safety profile, scalability, and clinical efficacy of CAR-T therapy. Recent advancements in biomarker-guided, antigen-specific CAR-T constructs and the incorporation of advanced imaging techniques could enable more precise and dynamically adjustable treatment protocols.
